# The Relationship between Nonalcoholic Fatty Liver Disease and Retinopathy in NHANES III

**DOI:** 10.1371/journal.pone.0165970

**Published:** 2016-11-01

**Authors:** Tzu-Yu Lin, Ying-Jen Chen, Wei-Liang Chen, Tao-Chun Peng

**Affiliations:** 1 Department of Ophthalmology, Shuang Ho Hospital, Taipei Medical University, New Taipei City, Taiwan; 2 Department of Ophthalmology, Tri-Service General Hospital, and School of Medicine, National Defense Medical Center, Taipei, Taiwan; 3 Division of Family Medicine, Department of Family and Community Medicine, Tri-Service General Hospital, and School of Medicine, National Defense Medical Center, Taipei, Taiwan; 4 Division of Geriatric Medicine, Department of Family and Community Medicine, Tri-Service General Hospital, and School of Medicine, National Defense Medical Center, Taipei, Taiwan; 5 Graduate Institute of Medical Sciences, National Defense Medical Center, Taipei, Taiwan; University of Catania, ITALY

## Abstract

**Background:**

Nonalcoholic fatty liver disease (NAFLD), an emerging multisystem disease, has the similar pathogenesis with diabetes and is prevalent in diabetes. This study investigated whether NAFLD is associated with retinopathy in individuals with diabetes and without diabetes.

**Methods:**

The association between NAFLD and retinopathy was investigated in 5963 participants aged 40 years and older who participated in the NHANES III, a nationally representative, population-based and cross-sectional study. NAFLD was detected via ultrasonography, and fundus photographs were obtained to grade retinopathy patterns. We performed multivariate logistic regression analysis to investigate the relationship between the presence of retinopathy and NAFLD and diabetes.

**Results:**

After adjusting for multiple covariates, NAFLD population had no evidence of retinopathy increase in population without diabetes (odds ratio [OR]: 0.77; 95% confidence interval [CI]: 0.48 to 1.26). In addition, NAFLD in individuals with diabetes was not significantly associated with retinopathy (OR: 0.77; 95% CI: 0.47 to 1.26), independent of age, gender, ethnicity, waist circumference, serum high-density lipoprotein (HDL) cholesterol, serum triglycerides, systolic blood pressure, and glycated hemoglobin.

**Conclusions:**

In the US general population, NAFLD is not a precipitating factor of retinopathy in population with or without diabetes.

## Introduction

Retinopathy is a type of retinal microvasculature disease and a well-known complication of diabetes and hypertension[1]. Most previous studies have investigated diabetic retinopathy (DR)-related issues because DR is a primary cause of blindness worldwide[2]. The prevalence of DR is approximately 33% in Western countries[3]. The key pathogenic mechanism of DR is hyperglycemia due to impaired insulin action as the result of insulin deficiency or insulin resistance[4]. In recent years, several reports have shown that nonalcoholic fatty liver disease (NAFLD) has the similar epidemiological and pathophysiological features with type 2 diabetes and metabolic syndrome[5–7]. Increasing evidence has demonstrated that NAFLD is associated with an increased prevalence of micro- and macrovascular complications in person with diabetes[8]. However, controversy remains regarding the relationship between NAFLD and retinopathy, particularly in individuals with diabetes[9–12]. The role of NAFLD in management of DR is not clear. In addition, there is scarce evidence regarding whether NAFLD is associated with retinopathy in individuals without diabetes. Therefore, this study aimed to explore the association between NAFLD and retinopathy in individuals with or without diabetes using the Third National Health and Nutrition Examination Survey (NHANES III).

## Materials and Methods

### Study population and data collection

The NHANES III was conducted by the Centers for Disease Control and Prevention using a nationwide probability sample of the United States non-institutionalized civilian population from 1988 to 1994[13]. The NHANES III survey data contain a wide age range. Participants visited the mobile examination center (MEC) or underwent a home examination. All suitable participants underwent a standardized interview questionnaire that included demographic, socioeconomic, dietary, and health-related questions, such as smoking history, alcohol use, medical history, and physical activity. The participants also underwent detailed medical and physiological measurements during the examinations and laboratory testing. The program was approved by the National Center for Health Statistics (NCHS) Institutional Review Board in accordance with the Declaration of Helsinki, and all participants in NHANES had written informed consent prior to the study. Because our analysis exclusively used de-identified data, it was exempt from IRB review.

### Fundus photography and retinopathy definition

An examiner at the MEC photographed the ocular fundus of a randomly selected eye in participants aged over 40 years. One non-stereoscopic, color, 45-degree photograph centered between the optic nerve and macula was obtained with a film-based Canon CR4–45NM non-mydriatic fundus camera (Canon USA, Lake Success, New York, USA). If an extremely small pupil, severe corneal or lens opacity, complete retinal detachment, or other prohibitive factors were observed in the randomly selected eye, the other eye was photographed. No photograph was obtained if neither eye was suitable.

The presence of DR, age-related maculopathy, and other retinal diseases was assessed using the fundus images by photograph graders from the University of Wisconsin-Madison, Department of Ophthalmology. Retinopathy was defined as the presence of the following factors on the fundus photograph: retinal microaneurysms only (level 20); hemorrhages, soft exudates, hard exudates, or intraretinal microvascular abnormalities (IRMAs) without microaneurysms (levels 14 and 15); non-proliferative DR (levels 31, 41, and 51); proliferative DR (levels 60, 65, and 70); or non-DR (level 12).

### NAFLD and other variables

Participants underwent a gallbladder ultrasound in NHANES III program. A hepatic steatosis examination was conducted to assess the presence of fat within the hepatic parenchyma. The archived original gallbladder ultrasonography videotapes and NHANES III results observed during the MEC examination were reviewed.

Hepatic steatosis was evaluated using the following five criteria: parenchymal brightness, liver to kidney contrast, deep beam attenuation, bright vessel walls, and gallbladder wall definition. The presence, absence, or degree of these five criteria was recorded. An experienced radiologist supervised the trained ultrasound image readers. Aside from normal sonography patterns, the degree of hepatic steatosis severity was classified as mild, moderate, or severe.

Diabetes was defined as a fasting plasma glucose level ≥126 mg/dL, glycated hemoglobin level ≥6.5%, diabetes history, or hypoglycemia medication use. A trained research assistant measured blood pressure. Waist circumference was measured by a steel measuring tape at the iliac crest from the right side of the body. Serum HDL cholesterol, triglycerides, and glycated hemoglobin were measured via chemical analyses (Hitachi 737 Analyzer; Indianapolis, Indiana)

### Statistical analyses

We classified the participants into four groups as follows: group 1, no diabetes mellitus (DM) or NAFLD; group 2, only NAFLD; group 3, only DM; and group 4, both DM and NAFLD. Retinopathy was considered as present or absent. The baseline characteristics of the participants were compared with a Pearson chi-square test for categorical variables or and ANOVA or Kruskal-Wallis test for continuous variables.

Multivariate logistic regression analysis was also performed to assess associations between the four groups and retinopathy. In addition, group 1 was considered as a reference group. Models were adjusted for demographic factors as follows: model 1 was adjusted for age; model 2 was additionally adjusted for gender and ethnicity; and model 3 was adjusted for waist circumference, serum HDL cholesterol, serum triglycerides, systolic blood pressure, and glycated hemoglobin.

Additional analyses were conducted in groups 3 and 4. For these analyses, group 3 was considered the reference group and the relationships between the two groups and retinopathy were investigated by multivariate logistic regression analysis. This analysis was also adjusted for the demographic factors included in models 1, 2, and 3. Moreover, the fundus patterns based on the NHANES III classification were compared between the two groups without diabetes. A p-value<0.05 was considered statistically significant. All analyses were performed using SPSS (Version 18.0 for Windows, SPSS, Inc., Chicago, IL, USA) and utilized the sample weights and design effects of the survey.

## Results

### Baseline characteristics

The numbers of participants in the four groups were as follows: group 1 (n = 3807), group 2 (n = 1211), group 3 (n = 486), and group 4 (n = 459) (Table 1). The mean age of participants was 54 year-old. Retinopathy was detected more frequently in the participants with diabetes (21.9% in group 3 and 19.1% in group 4). Higher glycated hemoglobin was noted in participants with NAFLD (5.32±0.02% in group 1 vs. 5.39±0.02% in group 2; and 7.37±0.18% in group 3 vs. 7.77±0.16% in group 4). Compared with the other three groups, group 4 also had a greater waist circumference, higher serum triglycerides, and higher systolic blood pressure.

**Table 1:** Clinical characteristics of diabetic and NAFLD individuals in the Third National Health and Nutrition Examination Survey population.

	Group				
Characteristic	Total Population (n = 5963)	DM(-)& NAFLD(-) (n = 3807)	DM(-)& NAFLD(+) (n = 1211)	DM(+)& NAFLD(-) (n = 486)	DM(+)& NAFLD(+) (n = 459)
Age (years)	54.03 ± 0.32	53.37± 0.35	54.27±0.39	57.77±0.66	58.11±0.80
Male (%)	46.2±0.8	43.7±0.9	54.7±1.5	43.4±3.5	48.5±4.2
Ethnicity (%)					
Non-Hispanic white	80.8±1.3	82.2±1.3	81.5±1.8	66.1±3.7	73.9±2.9
Systolic blood pressure (mmHg)	126.36±0.41	124.18±0.44	130.04±0.91	132.21±1.36	135.57±1.14
Waist circumference (cm)	95.74±0.33	91.91±0.29	104.08±0.58	101.35±1.11	109.38±1.05
Serum HDL cholesterol (mg/dL)	50.39±0.42	52.63±0.47	45.18±0.74	47.03±1.07	43.34±1.26
Serum triglycerides (mg/dL)	161.85±3.19	136.22±2.20	210.64±6.63	215.68±29.14	265.86±14.53
Glycated hemoglobin (%)	5.56±0.03	5.32±0.02	5.39±0.02	7.37±0.18	7.77±0.16
Retinopathy (%)	5.3±0.5	3.6±0.6	3.5±0.7	21.9±3.9	19.1±2.2

Data are presented as the means ± standard errors or percentages ± standard errors.

DM: diabetes mellitus

NAFLD: nonalcoholic fatty liver disease

HDL: high density lipoprotein

### NAFLD and DM

In the multivariate logistic regression analysis, retinopathy was significantly associated with the diabetes groups compared with group 1 (group 3: odds ratio [OR]: 3.84, 95% confidence interval [CI]: 2.50–5.92; group 4: OR: 2.66, 95% CI: 1.44–4.90) after adjusting for age, gender, ethnicity, waist circumference, serum HDL cholesterol, serum triglycerides, systolic blood pressure, and glycated hemoglobin (Table 2). However, no obvious relationship with retinopathy was observed in group 2 in the unadjusted model and models 1, 2, and 3. Exploratory results regarding the association between retinopathy and NAFLD in the groups with diabetes are shown in Table 3. Compared with group 3, NAFLD in the population with diabetes was not significantly associated with retinopathy in the unadjusted model and models 1, 2, and 3.

**Table 2:** Logistic regression analysis showing associations of retinopathy between four groups.

	Group			
	DM(-)& NAFLD(-)	DM(-)& NAFLD(+)	DM(+)& NAFLD(-)	DM(+)& NAFLD(+)
Unadjusted	1	0.97(0.58–1.63)	7.53(4.66–12.16)	6.33(3.89–10.31)
Model 1	1	0.96(0.57–1.60)	7.07(4.26–11.73)	5.91(3.55–9.85)
Model 2	1	0.94(0.56–1.56)	7.07(4.15–12.05)	5.86(3.53–9.74)
Model 3	1	0.77(0.48–1.26)	3.84(2.50–5.92)	2.66(1.44–4.90)

Data are presented as odds ratios (95% confidence intervals)

DM: diabetes mellitus

NAFLD: nonalcoholic fatty liver disease

Model 1: adjusted for age

Model 2: adjusted for age, gender, and race-ethnicity

Model 3: adjusted for age, gender, race-ethnicity, waist circumference, serum HDL cholesterol, serum triglycerides, systolic blood pressure, and glycated hemoglobin

**Table 3:** Logistic regression analysis showing associations of retinopathy between two groups in the diabetic population.

	Group		
	DM(+)& NAFLD(-)	DM(+)& NAFLD(+)	P
Unadjusted	1	0.84(0.57–1.23)	0.369
Model 1	1	0.84(0.57–1.24)	0.368
Model 2	1	0.84(0.56–1.24)	0.359
Model 3	1	0.77(0.47–1.26)	0.284

Data are presented as odds ratios (95% confidence intervals)

DM: diabetes mellitus

NAFLD: nonalcoholic fatty liver disease

Model 1: adjusted for age

Model 2: adjusted for age, gender, and race-ethnicity

Model 3: adjusted for age, gender, race-ethnicity, waist circumference, serum HDL cholesterol, serum triglycerides, systolic blood pressure, and glycated hemoglobin

### Fundus photographs in the four groups

The characteristics of the fundus photographs stratified by retinopathy status are shown in Table 4. In the groups with diabetes, early-, moderate-, and severe-stage non-proliferative retinopathy were the most commonly observed and no matter with NAFLD or not. In the groups without diabetes, microaneurysms were most commonly observed, followed by non-proliferative retinopathy and hemorrhage with NAFLD only. Non-proliferative retinopathy were observed, but no proliferative retinopathy was noted. No obvious differences in fundus images were observed between the population groups without diabetes.

**Table 4:** Different retinopathy patterns in four groups.

Retinopathy Lesion	DM(-)& NAFLD(-)	DM(-)& NAFLD(+)	DM(+)& NAFLD(-)	DM(+)& NAFLD(+)
MA only	61.1	65.8	40.9	30.5
Hemorrhage, hard/soft exudates, IRMA without MA	20.8	15.6	8.8	15.1
Nonproliferative retinopathy	10.5	12.1	44.9	45.4
Nondiabetic retinopathy	7.6	6.5	2.3	4.8
Proliferative retinopathy	0	0	3.1	4.2

IRMA: intraretinal microvascular abnormalities

MA: microaneurysms

DM: diabetes mellitus

NAFLD: nonalcoholic fatty liver disease

## Discussion

In this study, a strong association between diabetes and retinopathy was observed as previous reports[2–4]. However, we observed that NAFLD was not associated with retinopathy in a population without diabetes. Additionally, NAFLD was not associated with an increased prevalence of retinal disease in person with diabetes. No significant relationship between NAFLD and retinopathy was observed in the person with diabetes and without diabetes after adjusting for the confounding factors of age, gender, ethnicity, and metabolic components.

Until now, few studies have addressed the relationship between NAFLD and retinopathy in the presence of diabetes, and there are conflicting results. Kim et al.[9] reported that NAFLD is inversely associated with DR prevalence in Korean people with type 2 diabetes. Leite et al. noted that lower prevalence of retinopathy in ultrasound-diagnosed NAFLD [10] or biopsy proven NAFLD [14] adults with diabetes in Brazil. In contrast, Liccardo et al.[11] observed a positive association between pediatric NAFLD participants and the degree of retinopathy signs. Targher et al.[12] suggested that NAFLD is associated with an increased prevalence of proliferative/laser-treated retinopathy in individuals with type 2 diabetes in Italy. These heterogeneous results may have been due to the variety of ethnic populations and different age distributions. While the population studied by Targher et al.[12] included participants undergoing regular return visits to an outpatient clinic in Italy, our study performed in a nationally representative sample of US men and women. The percentage of concurrent NAFLD and DR participants was much higher in the Italy sample compared with the NHANES population assessed here (50% vs. 19.1%). In addition, NAFLD and non-proliferative retinopathy (NPDR) were only weakly associated after adjusting for multiple factors, as mentioned by Targher et al. As estimated by our study population, more than 90% of participants with diabetes had NPDR. The link between NAFLD and NPDR was weak which is in accordance with the results of Targher et al. However, we could not definitively corroborate the association between NAFLD and PDR due to the small population of proliferative diabetic retinopathy (PDR) people.

Different patterns of retinopathy may be induced by different diseases, such as generalized or focal retinal arteriolar narrowing and arteriovenous nicking related to high blood pressure[15]. In addition to hypertension, Hubbard et al.[16] found that generalized arteriolar narrowing was related to systemic markers of inflammation. Nevertheless, high blood sugar level is undoubtedly a powerful predisposing factor for retinopathy[17]. Abnormal glucose metabolic pathway activity is the primary contributor to the structural and physiological effects on retinal capillaries, which become both functionally and anatomically incompetent[18]. The pathophysiological mechanism of NAFLD, as discussed previously, is recognized as the condition of insulin resistance, the hepatic manifestation of metabolic syndrome[5]. NAFLD has been associated with an increased risk of diabetes mellitus in different populations [19,20] and, thus, has been potentially associated with an increased risk of retinopathy. However, in our study, no synergistic effect of NAFLD on retinopathy development was observed in the diabetes groups even though the participants with NAFLD indeed tended to have higher glycated hemoglobin. Although the exact reason for this remains unclear, one plausible explanation is that NAFLD has different pathophysiological influence on retinopathy apart from hyperglycemia.

As for the participants without diabetes, there are other potential explanations for the non-significant relationship between NAFLD and retinopathy. The characteristics of NALFD and metabolic syndrome are similar[21,22]. Metabolic syndrome is believed to decrease insulin effects due to insulin resistance, thus influencing the function to suppress plasma free fatty acids[23]. The accumulation of fatty acids in the liver may cause a fatty liver. In a previous study, Jeremy et al.[24] found no evidence of an association between metabolic syndrome and non-diabetic retinopathy. In the Atherosclerosis Risk in Communities (ARIC) study, which was based in the United States, no relationship between metabolic syndrome and retinopathy was observed in individuals without diabetes[25]. In recent study, Yan et al.[26] reported that NAFLD combined with type 2 diabetes group had a lower prevalence of diabetic retinopathy than type 2 diabetes-alone group. However, Yang et al.[27] noted that NAFLD is a predisposing factor for retinal artery lesions, but individuals with diabetes may have been included in this previous study and confounded the result. Therefore, high blood pressure and hyperglycemia appear to be the key risk factors for retinopathy and may explain the pathogenesis better than metabolic syndrome independently. Thus, our results support the notion that NAFLD, a feature of metabolic syndrome[28,29], is also not significantly associated with retinopathy in persons without diabetes.

This study has some limitations. First, only one eye was photographed in the NHANES III population, which may have caused an underestimation of the prevalence of retinopathy. Second, this study was cross-sectional; thus, we were unable to ascertain the order of onset for each event and the importance of this order. Whether there is a permanent or transient relationship between NAFLD and retinopathy is unknown. Third, ultrasonography was used to examine the national prevalence of NAFLD instead of a biopsy. Although the sensitivity and specificity differences between ultrasonography and liver biopsy are small[30], ultrasonography may still assess dead space, thereby affecting results.

Although NAFLD is viewed as a type of metabolic syndrome, no additional effects on retinopathy were observed in the group with diabetes, and, therefore it should not be considered a precipitating factor of retinopathy in the US general population. Further prospective and longitudinal studies and investigations with different demographic populations will aid in understanding the role of NAFLD in populations with or without diabetic.
